# Hemostatic compression treatment of central placenta previa with modified Cho suture

**DOI:** 10.1093/jscr/rjaf880

**Published:** 2025-11-11

**Authors:** Paolo Meloni, Sara Izzo, Terenzia Simari, Andrea Polistena, Daniela Messineo, Pierfrancesco Di Cello, Silvia Lai, Luciano Izzo, Marcello Molle, Paolo Izzo

**Affiliations:** SC Ostetricia Ginecologica, Ospedale Imperia, ASL 1, Strada Calderina-Imperia 1, 18013 Diano Marina IM, Italy; Plastic and Reconstructive Surgery Unit, Multidisciplinary Department of Medical-Surgical and Dental Specialties, Università degli Studi della Campania “L.Vanvitelli”, Piazza Luigi Miraglia 2, 80138 Naples, Italy; Ambulatorio Specialistico Ginecologia Ostetricia, ASL 1, Strada Calderina-Imperia 1, 18013 Diano Marina IM, Italy; “Pietro Valdoni” Department of Surgery, Policlinico “Umberto I”, “Sapienza” University of Rome, Viale del Policlinico 155, 00161, Rome, Italy; Department of Radiological, Oncological, and Anatomical Pathology Sciences, La Sapienza University, Viale del Policlinico 155, 00161 Rome, Italy; UOC General Surgery Frosinone-Alatri, ASL Frosinone, Frosinone, Italy; Department of Translational and Precision Medicine, Nephrology Unit, Policlinico “Umberto I”, “Sapienza” University of Rome, Viale del Policlinico 155, 00161 Rome, Italy; “Pietro Valdoni” Department of Surgery, Policlinico “Umberto I”, “Sapienza” University of Rome, Viale del Policlinico 155, 00161, Rome, Italy; Plastic and Reconstructive Surgery Unit, Multidisciplinary Department of Medical-Surgical and Dental Specialties, Università degli Studi della Campania “L.Vanvitelli”, Piazza Luigi Miraglia 2, 80138 Naples, Italy; “Pietro Valdoni” Department of Surgery, Policlinico “Umberto I”, “Sapienza” University of Rome, Viale del Policlinico 155, 00161, Rome, Italy

**Keywords:** placenta previa, modified Cho suture, postpartum hemorrhage, hemostatic uterine compression, cesarean section management

## Abstract

Placenta previa (PP) occurs when the placental edge lies within 2 cm of the internal cervical orifice and increases the risk of placenta accreta, especially in women with prior cesarean delivery, uterine surgery, or advanced maternal age. Patients with central PP typically undergo elective cesarean section. Hemorrhagic complications are common even in the absence of accreta. We present the case of a 46-year-old woman who conceived through in vitro fertilization and with an uneventful pregnancy. A third-trimester ultrasound confirmed central PP. An elective cesarean section was scheduled at 38 weeks. Manual removal of the placenta was difficult, and bleeding from the placental bed persisted. Due to the midline location of the bleeding vessels, modified Cho compression sutures were applied solely to the posterior uterine wall using resorbable monofilament, achieving effective hemostasis. This case highlights that modified compression sutures can be adapted to control hemorrhage effectively, reducing the need for more invasive interventions.

## Background and introduction

A placenta is defined as previa when the placental edge does not cover the internal orifice but is located within 2 cm of it. The incidence of placenta previa (PP) is ~5/1000 births [1]. If PP is diagnosed during the early stages of pregnancy, it usually resolves by the 28th week due to the enlargement of the uterus. According to the Royal College of Obstetricians and Gynaecologists (RCOG) classification, PP is classified echographically according to its clinical significance: if the placenta completely covers the internal uterine orifice (IUO), it is considered major PP (formerly complete and partial central PP); if the placental edge lies on the IUO but does not cover the IUO, it is referred to as minor PP (formerly marginal and lateral PP) [2]. Transvaginal ultrasound (TVU-2D) is the gold standard for diagnosing PP. If PP is suspected on abdominal ultrasound performed at 20–22 weeks, it must then be confirmed with a TVU [3, 4]. PP is a risk factor for placental accreta, which is pathological adhesion to the uterus due to a defect in the basal decidua with invasion of the myometrium by chorionic villi. In the absence of risk factors, the incidence of accreta in cases of PP is ~1 case per 22 000 births [5]. The risk of accreta also increases if risk factors are present: previous cesarean delivery, maternal age over 35 years, previous uterine surgery, and multiparity [6, 7]. In cases of suspected asymptomatic major PP or in cases of doubtful accreta, a further transabdominal ultrasound (ETA) and ETV should be performed at 32 weeks of gestation to clarify the diagnosis with any further tests, adequate counseling should be provided, and an adequate team should be available to plan the delivery in an appropriate facility [8].

For patients with PP or low insertion, risks include abnormal fetal presentation, premature rupture of membranes, fetal growth restriction, vasa previa, and velamentous insertion of the umbilical cord (in which the placental end of the cord consists of divergent umbilical blood vessels surrounded only by fetal membranes). In women who have had a previous cesarean delivery, PP increases the risk of placenta accreta; the risk increases significantly with the number of previous cesarean deliveries (from ~6%–10% for one cesarean delivery to >60% for >4) [9, 10]. Patients with central PP undergo elective cesarean delivery. The afterbirth can cause significant bleeding even in the absence of accreta. Compressive uterine sutures are conservative surgical procedures used as a second-line treatment to control severe post-partum hemorrhages and avoid hysterectomy [11, 12]. The compressive hemostatic suture in these cases is according to Cho’s suture [13], which involves the use of a straight needle and 0-gauge monofilament to compress the anterior and posterior walls of the uterus with simple stitches that pierce the uterus through its entire thickness.

## Case presentation

**Figure 1:**
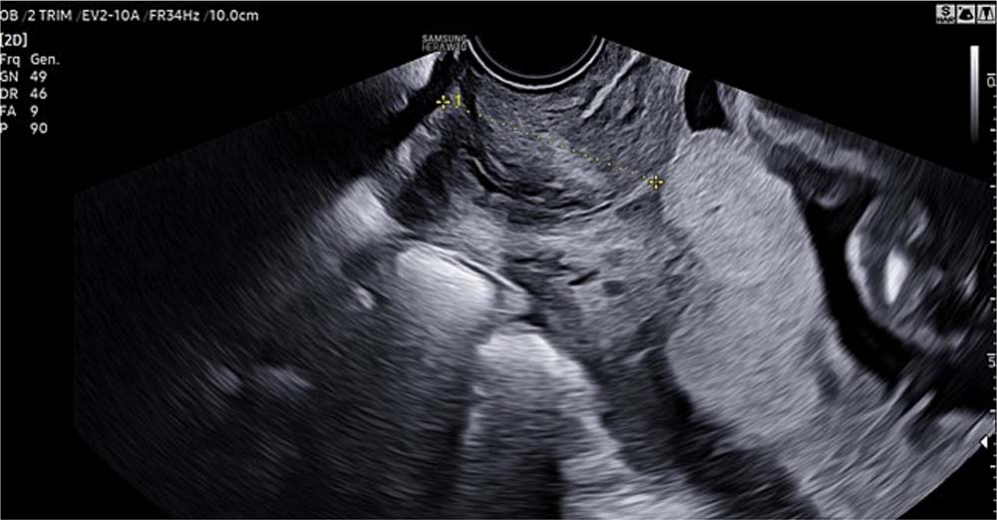
TVU confirming diagnosis of central PP.

A 46-year-old woman underwent in vitro fertilization (IVF). Pregnancy progressed normally, with physiological development and appropriate fetal growth. In the third trimester, an ultrasound confirmed the diagnosis of a central PP (Fig. 1).

A cesarean section was planned. In preparation, units of packed red blood cells, plasma, and coagulation factors were arranged, and the transfusion center and blood bank were notified due to the risk of potential massive hemorrhage. The surgery was performed electively at 38 weeks of gestation, considering the additional risk factor of advanced maternal age. The newborn was in excellent condition at birth, with an Apgar score of 9/10 and an umbilical cord pH of 7.3.

Manual delivery of the placenta proved challenging, particularly in the central isthmic region covering the internal cervical os. The uterus was exteriorized, and careful removal of the placenta, followed by curettage of the implantation site, was performed. Although the uterus appeared well-contracted, bleeding persisted from the placental bed. A marker was placed in the cervical canal to prevent closure during suture.

Because the bleeding vessels were located along the midline of the placental bed, standard Cho sutures were deemed unsuitable, as they would require bringing the anterior and posterior uterine walls together—risking closure of the cervical canal. Instead, modified Cho compression sutures were applied exclusively to the posterior isthmic wall. One suture was placed at the cervical level, and the second just above it. A resorbable monofilament thread was used.

**Figure 2:**
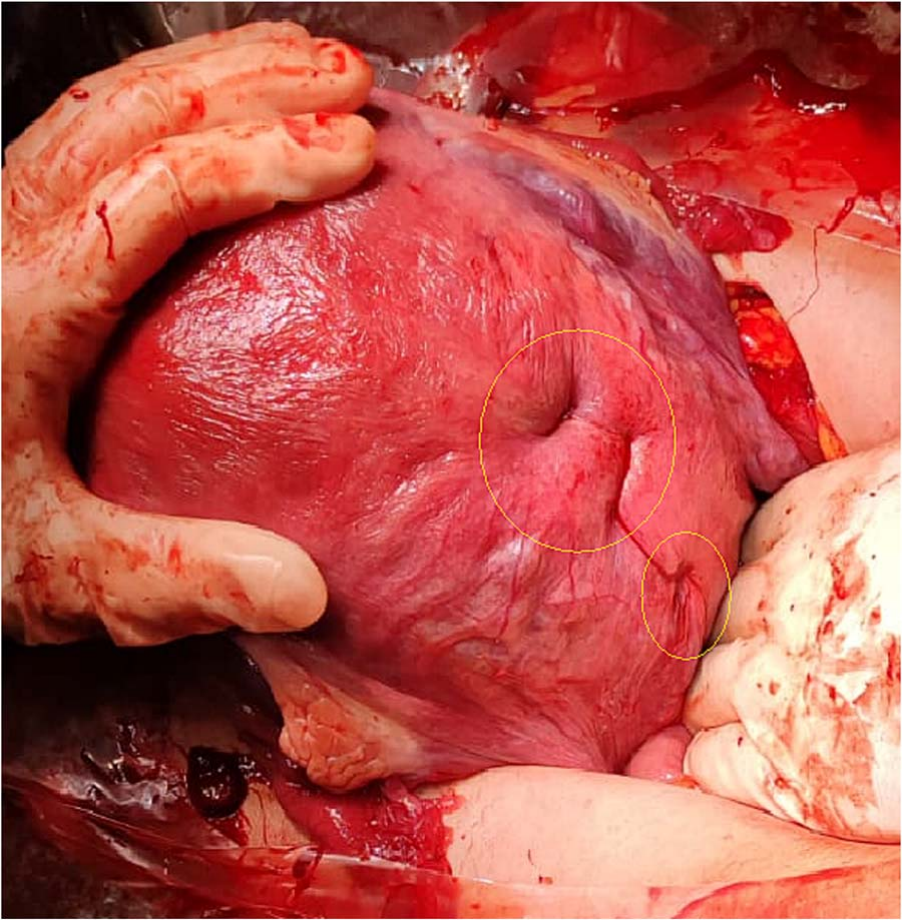
Hemostatic suture with modified Cho stitches on the posterior wall and cervical isthmus.

The suture technique involved passing the needle through the uterine cavity from anterior to posterior, exiting the posterior wall, and reinserting it 2–3 cm laterally from posterior to anterior. The needle was then passed again from anterior to posterior, exiting the posterior wall ~2–3 cm below the previous exit site. Finally, the stitch was completed by reinserting 2–3 cm laterally into the posterior wall. The two ends of the thread were tied using a flat surgical knot to enhance compression. Both sutures were placed medially along the posterior wall at the level of the cervical isthmus (Fig. 2).

## Discussion and conclusion

In the clinical case described, several risk factors could significantly impact maternal and fetal outcomes (advanced maternal age, the presence of PP, and its central location.

Since mass screening for abnormal placentation is not feasible, it is crucial to focus on in-depth evaluation of at-risk populations—those with PP, prior uterine surgery or curettage, or advanced maternal age. Familiarity with the available diagnostic tools is essential. Improved maternal-fetal outcomes are achievable through accurate prenatal diagnosis and a coordinated multidisciplinary approach during delivery.

TVU remains the most reliable diagnostic method for identifying PP in all its forms [14]. Surgical management should be entrusted to an experienced team, particularly because the risk of placenta accreta exists even in patients without a history of cesarean delivery, the primary known risk factor.

Compressive sutures are highly effective in managing uterine bleeding and promoting contractility. In this case, a modified Cho suture was used to achieve hemostasis along the median plane of the posterior uterine wall. This demonstrates that, in expert hands, compression sutures can be adapted to the specific challenges of the clinical situation while preserving their effectiveness.

## Data Availability

The datasets generated and analyzed during the current study are not publicly available due to concerns regarding participant anonymity. Requests for access to the data should be directed to the corresponding author.
